# The effects of *URAT1/SLC22A12* nonfunctional variants,R90H and W258X, on serum uric acid levels and gout/hyperuricemia progression

**DOI:** 10.1038/srep20148

**Published:** 2016-01-29

**Authors:** Masayuki Sakiyama, Hirotaka Matsuo, Seiko Shimizu, Hiroshi Nakashima, Takahiro Nakamura, Akiyoshi Nakayama, Toshihide Higashino, Mariko Naito, Shino Suma, Asahi Hishida, Takahiro Satoh, Yutaka Sakurai, Tappei Takada, Kimiyoshi Ichida, Hiroshi Ooyama, Toru Shimizu, Nariyoshi Shinomiya

**Affiliations:** 1 Department of Integrative Physiology and Bio-Nano Medicine, National Defense Medical College, 3-2 Namiki, Tokorozawa, Saitama 359-8513, Japan; 2 Department of Dermatology, National Defense Medical College, 3-2 Namiki, Tokorozawa, Saitama 359-8513, Japan; 3 Department of Preventive Medicine and Public Health, National Defense Medical College, 3-2 Namiki, Tokorozawa, Saitama 359-8513, Japan; 4 Laboratory for Mathematics, National Defense Medical College, 3-2 Namiki, Tokorozawa, Saitama 359-8513, Japan; 5 Department of Preventive Medicine, Nagoya University Graduate School of Medicine, 65 Tsurumai-cho, Showa-ku, Nagoya, Aichi 466-8550, Japan; 6 Department of Pharmacy, The University of Tokyo Hospital, Faculty of Medicine, The University of Tokyo, 7-3-1 Hongo, Bunkyo-ku, Tokyo 113-8655, Japan; 7 Department of Pathophysiology, Tokyo University of Pharmacy and Life Sciences, 1432-1 Horinouchi, Hachiouji, Tokyo 192-0392, Japan; 8 Ryougoku East Gate Clinic, 3-21-1 Ryougoku, Sumida-ku, Tokyo 130-0026, Japan; 9 Midorigaoka Hospital, 3-13-1 Makami-cho, Takatsuki, Osaka 569-1121, Japan; 10 Kyoto Industrial Health Association, 67 Kitatsuboi-cho, Nishinokyo, Nakagyo-ku, Kyoto 604-8472, Japan

## Abstract

Urate transporter 1 (*URAT1/SLC22A12*), a urate transporter gene, is a causative gene for renal hypouricemia type 1. Among several reported nonsynonymous *URAT1* variants, R90H (rs121907896) and W258X (rs121907892) are frequent causative mutations for renal hypouricemia. However, no case-control study has evaluated the relationship between gout and these two variants. Additionally, the effect size of these two variants on serum uric acid (SUA) levels remains to be clarified. Here, 1,993 primary gout patients and 4,902 health examination participants (3,305 males and 1,597 females) were genotyped with R90H and W258X. These *URAT1* variants were not observed in any gout cases, while 174 subjects had the *URAT1* variant in 2,499 health examination participants, respectively (*P* = 8.3 × 10^−46^). Moreover, in 4,902 health examination participants, the *URAT1* nonfunctional variants significantly reduce the risk of hyperuricemia (*P* = 6.7 × 10^−19^; risk ratio = 0.036 in males). Males, having 1 or 2 nonfunctional variants of *URAT1*, show a marked decrease of 2.19 or 5.42 mg/dl SUA, respectively. Similarly, females, having 1 or 2 nonfunctional variants, also evidence a decrease of 1.08 or 3.89 mg/dl SUA, respectively. We show that *URAT1* nonfunctional variants are protective genetic factors for gout/hyperuricemia, and also demonstrated the sex-dependent effect size of these *URAT1* variants on SUA (*P* for interaction = 1.5 × 10^−12^).

Gout (MIM 138900) is one of the most common types of inflammatory arthritis as a consequence of hyperuricemia. Gout and hyperuricemia increase the risk of other common diseases, such as kidney diseases, cerebrovascular diseases, hypertension and cardiovascular diseases^1^. Several transporter genes associated with gout and serum uric acid (SUA) levels were previously reported, such as ATP-binding cassette transporter, subfamily G, member 2 (*ABCG2/BCRP* [MIM 603756])^2^,^3^,^4^,^5^,^6^, glucose transporter 9 (*GLUT9/SLC2A9* [MIM 606142])^2^,^7^,^8^, sodium-dependent phosphate cotransporter type 1 (*NPT1/SLC17A1* [MIM 182308])^9^, organic anion transporter 4 (*OAT4/SLC22A11* [MIM 607097])^10^,^11^, and urate transporter 1 (*URAT1/SLC22A12* [MIM 607096])^12^,^13^.

Among them, *URAT1*, which is a well-known urate transporter gene, has been identified as a causative gene for renal hypouricemia type 1 (MIM 220150)^14^. Among several reported nonsynonymous variants in *URAT1*, R90H (rs121907896) and W258X (rs121907892) are frequent causative mutations for renal hypouricemia^15^. Previous *in vitro* functional studies showed that R90H variant diminishes the urate transport activity of URAT1^15^ as the other common variant, W258X^14^. It has been also reported that nonfunctional variants in *URAT1* were not detected in 77 Spanish gout patients^16^, and W258X in *URAT1* suppressed the development of gout^17^. However, to our knowledge, no large-scale case-control study has evaluated the relationship between gout/hyperuricemia and both variants (R90H and W258X). In this study, therefore, we investigated the association between gout and two *URAT1* variants with large-scale Japanese primary gout cases and controls. Moreover, the risk ratio (RR) of these two nonfunctional variants for hyperuricemia was evaluated in approximately 5,000 Japanese health examination participants. Although there is a gender difference in SUA due to sex hormones^18^,^19^, the effect size of these two variants on SUA in each sex remains to be clarified. Furthermore, these *URAT1* variants (R90H and W258X) are frequently observed especially in a Japanese population; thus, it is particularly important to analyze the sex-dependent effect size of these *URAT1* variants on SUA in a general Japanese population. Therefore, we also evaluated the effect size of these *URAT1* variants on SUA in each sex with a large number of Japanese health examination participants.

## Results

### Case-control study of gout

The genotyping results of *URAT1* nonfunctional variants (R90H and W258X) for 1,993 gout cases and 2,499 controls were shown in Table 1. The two variants were in Hardy-Weinberg equilibrium (*P* > 0.05). The *URAT1* nonfunctional variants (R90H and W258X) were not observed in any gout cases (n = 1,993), while R90H heterozygotes (G/A), W258X heterozygotes (G/A) and W258X homozygotes (A/A) were observed in 22, 150 and 2 subjects, respectively, among 2,499 control subjects (*P* = 8.3 × 10^−46^; Table 1). This result is compatible with previous studies^16^,^17^, and indicates that these *URAT1* variants are protective factors of gout.

### Risk ratio for hyperuricemia

Next, Table 2 and Supplementary Table S1 show the genotype distributions of *URAT1* nonfunctional variants in 4,902 health examination participants of the Japan Multi-Institutional Collaborative Cohort (J-MICC) study (3,305 males and 1,597 females). Among the 4,902 participants, the nonfunctional allele frequencies of R90H and W258X were 0.28% and 2.24%, respectively. All of the participants were divided into hyperuricemia (SUA > 7.0 mg/dl) or control (SUA ≤ 7.0 mg/dl).

In 3,305 males (Table 2), there were significant differences between hyperuricemia and control in both R90H (*P* = 4.3 × 10^−3^) and W258X genotype distributions (*P* = 3.3 × 10^−16^). Additionally, the number of R90H or W258X nonfunctional alleles in each group was calculated. Then, the proportion of nonfunctional alleles was more frequent in control than that in hyperuricemia (*P* = 6.7 × 10^−19^; RR = 0.036; 95% confidence interval [CI]: 0.009–0.143, Table 2).

In 1,597 females, both R90H and W258X were observed only in controls (Supplementary Table S1). However, because the female hyperuricemia group was comprised of very small sample size (24 individuals), no association analysis was performed.

### Effect size of *URAT1* variants on SUA levels

**Figure 1:**
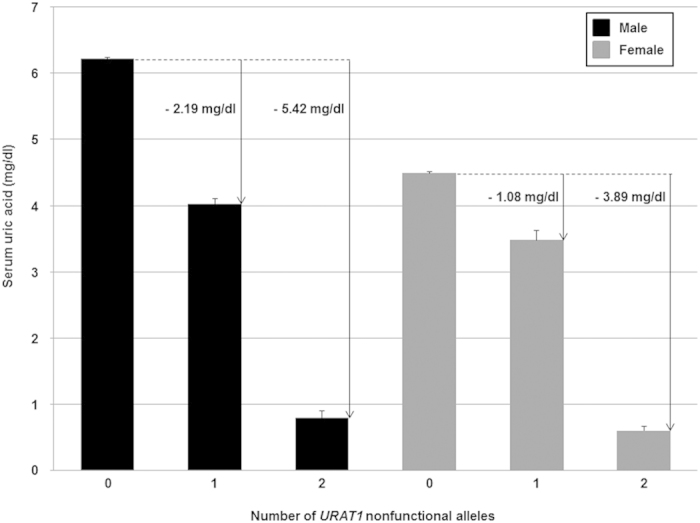
Changes of serum uric acid levels of *URAT1* nonfunctional alleles in health examination participants. 4,753 health examination participants, who received no medication for gout and/or hyperuricemia, were analyzed. Among 3,158 male participants (left, black bars), 0, 1 and 2 nonfunctional alleles (R90H or W258X) were detected in 2,982, 174 and 2 males, respectively. Among 1,595 female participants (right, grey bars), 0, 1 and 2 nonfunctional alleles (R90H or W258X) were detected in 1,529, 63 and 3 females, respectively. Serum uric acid (SUA) levels of participants having 0, 1 and 2 nonfunctional alleles were shown in each sex. The sex-dependent effect size of SUA decrease by nonfunctional alleles (arrow) was also shown. All bars are expressed as means ± SEM.

We also investigated SUA for each number of *URAT1* nonfunctional alleles using 4,753 individuals (3,158 males and 1,595 females), who received no medication for gout and/or hyperuricemia among 4,902 health examination participants of the J-MICC Study. The mean SUA levels with standard error of the mean (SEM) of having 0, 1 and 2 nonfunctional alleles were 6.22 ± 0.02, 4.03 ± 0.07 and 0.80 ± 0.10 mg/dl in males, respectively, and were 4.49 ± 0.02, 3.48 ± 0.15 and 0.60 ± 0.06 mg/dl in females, respectively (Fig. 1). Then, the nonfunctional alleles of two *URAT1* variants significantly decreased SUA in both males and females (*P* = 2.2 × 10^−138^ and 2.6 × 10^−24^, respectively).

Furthermore, a multiple regression analysis, which focused on the statistical significance of the interaction term, revealed that there was an interaction between *URAT1* nonfunctional variants and sex (*P* for interaction = 1.5 × 10^−12^, Table 3).

## Discussion

URAT1 has been identified as a urate-anion exchanger which regulates SUA levels by playing an important role in the reabsorption of urate in human kidney^14^. In this study, we performed the genotyping of the two *URAT1* nonfunctional variants (R90H and W258X), and demonstrated the association with gout (Table 1), and the significant effect on hyperuricemia progression (Table 2) and that on SUA (Fig. 1).

Consistent with previous reports (on 77 Spanish^16^ and 185 Japanese^17^ gout patients, respectively), no *URAT1* nonfunctional variants (R90H or W258X) were found even in our large number of gout patients (n = 1,993). Our results indicate that these *URAT1* variants prevent the development of gout by the large-scale case-control study (case = 1,993 and control = 2,499).

Moreover, we revealed that the *URAT1* nonfunctional alleles of R90H and W258X markedly reduce the risk of hyperuricemia (RR = 0.036 in males; Table 2) and severely decrease SUA (Fig. 1) using 4,902 health examination participants. Males, having 1 or 2 nonfunctional alleles of *URAT1* exhibit a marked decrease of 2.19 or 5.42 mg/dl SUA, respectively (Fig. 1). Similarly, females, having 1 or 2 nonfunctional alleles of *URAT1* also show a decrease of 1.08 or 3.89 mg/dl SUA, respectively (Fig. 1). Moreover, the interaction between *URAT1* nonfunctional variants and sex was present (*P* for interaction = 1.5 × 10^−12^, Table 3). Thus, for the first time, we demonstrated the sex-dependent effect size of SUA by *URAT1* nonfunctional variants, which is also important for understanding the pathogenesis of renal hypouricemia because mild renal hypouricemia (SUA ≤ 3.0 mg/dl) could be caused by a heterozygous nonfunctional variant of *URAT1*^20^ or *GLUT9*^21^. Our data clearly demonstrated that some individuals with a heterozygous *URAT1* nonfunctional variant exhibit renal hypouricemia.

Interestingly, although the sex difference in SUA is well-known^18^,^19^, SUA of the individuals having 2 nonfunctional alleles is similar between males (0.80 mg/dl) and females (0.60 mg/dl). Moreover, the sex difference in SUA is smaller in the individuals having 1 nonfunctional allele (0.55 mg/dl) than in individuals without nonfunctional alleles (1.73 mg/dl). In other words, our data show that the sex difference of SUA becomes greater as the number of functional alleles (wild-type alleles) of *URAT1* increases, which suggests that the presence of functional URAT1 transporter is strongly related to the sex difference in SUA.

Previously, the sex difference in the expression of URAT1 had been found in a mouse model^22^. In addition, testosterone reportedly enhances the mRNA of Urat1 in a mouse model^23^ and increases promoter activity of human *URAT1*^24^. Combined with these previous reports, our data suggest that one of the main causes of the sex difference in SUA is the different expression levels of functional URAT1 transporters between males and females due to sex hormones.

In summary, we demonstrated that the *URAT1* nonfunctional variants are protective genetic factors for gout and hyperuricemia, and showed the sex-dependent effect size of these *URAT1* variants on SUA. These findings provide a better understanding of genetic factors for SUA and gout/hyperuricemia progression.

## Methods

### Patients and controls

This study was approved by the institutional ethical committee of the National Defense Medical College. All procedures were performed in accordance with the Declaration of Helsinki, and written informed consent was obtained from each subject participating in the present study.

In a case-control study of gout, 1,993 Japanese male patients with primary gout were recruited from the outpatients of Midorigaoka Hospital (Osaka, Japan), Kyoto Industrial Health Association (Kyoto, Japan) and Ryougoku East Gate Clinic (Tokyo, Japan). All of the gout patients were diagnosed according to the criteria established by the American College of Rheumatology^25^. Hyperuricemia was defined as the SUA level that exceeds 7.0 mg/dl (=416.36 mol/l) according to the guideline of the Japanese Society of Gout and Nucleic Acid Metabolism^26^. As the control group, 2,499 male Japanese individuals without hyperuricemia and gout history were selected from participants in the Shizuoka area in the J-MICC Study^27^,^28^.

For evaluation of the influence of two *URAT1* variants on SUA, 4,902 Japanese individuals (3,305 males including above 2,499 controls, and 1,597 females) were also recruited from health examination participants in the J-MICC Study. The details of participants in this study are shown in Supplementary Tables S2 and S3.

### Genotyping

Genomic DNA was extracted from whole peripheral blood cells^21^. Genotyping of R90H and W258X variants in *URAT1* was performed by TaqMan method (Life Technologies Corporation, Carlsbad, CA, USA) with a LightCycler 480 (Roche Diagnostics, Mannheim, Germany)^29^. Custom TaqMan assay probes were designed as follows: for R90H in *URAT1*, VIC-CCGCCACTTCCGC and FAM-CGCCGCTTCCGC; for W258X in *URAT1*, VIC-CGGGACTGAACACTG and FAM-CGGGACTGGACACTG. All of R90H heterozygotes (G/A), W258X heterozygotes (G/A) and W258X homozygotes (A/A) were confirmed by direct sequencing with a 3130xl Genetic Analyzer (Life Technologies Corporation)^29^ and the following primers: for R90H in *URAT1*, forward 5′-GTTGGAGCCACCCCAAGTGAC-3′ and reverse 5′-GTCTGACCCACCGTGATCCATG-3′; for W258X in *URAT1*, forward 5′-TGATGAACACGGGCACTCTC-3′ and reverse 5′-CTTTCCACTCGCTCCCCTAG-3′.

### Data analysis

For all calculations in the statistical analysis, the software R (version 3.1.1) (http://www.r-project.org/) was used^30^. The association analyses were examined with the Fisher’s exact tests. RRs were calculated under a dominant model: i.e. G/G versus G/A or A/A in W258X, 0 versus 1 or 2 in the number of nonfunctional alleles, respectively. Linear regression analyses were performed to evaluate the influence of two *URAT1* variants on SUA. Furthermore, we carried out a multiple regression analysis with an interaction term (*x*_*1*_* *x**_*2*_): *y* = *b*_*0*_ + *b*_*1*_* *x**_*1*_ + *b*_*2*_* *x**_*2*_ + *b*_*3*_*x*_*1*_* *x**_*2*_, where *y* is SUA level, *x*_*1*_ is an ordinal variable representing the number f nonfunctional alleles of two *URAT1* variants, and *x*_*2*_ is a dummy variable representing the sex (male = 0 and female = 1). For the robustness of the statistical test, random re-sampling methods with computer simulation are often applied^31^,^32^. In this study, the permutation test^32^ was used for random re-sampling in a case-control study with replacement for 1,000,000 times, and the robustness of statistics was confirmed. All *P* values were two-tailed and *P* value < 0.05 was considered statistically significant.

## Additional Information

**How to cite this article**: Sakiyama, M. *et al.* The effects of *URAT1/SLC22A12* nonfunctional variants, R90H and W258X, on serum uric acid levels and gout/hyperuricemia progression. *Sci. Rep.* **6**, 20148; doi: 10.1038/srep20148 (2016).

## Supplementary Material

Supplementary Information

## Figures and Tables

**Table 1:** Genotype distributions of nonfunctional variants in *URAT1/SLC22A12* in gout patients and controls.

	Number	R90H			W258X			Number of *URAT1* nonfunctional alleles*			
		G/G	G/A	A/A	G/G	G/A	A/A	0	1	2	*P* value†
Gout	1,993	1,993	0	0	1,993	0	0	1,993	0	0	
Control	2,499	2,477	22	0	2,347	150	2	2,325	172	2	8.3 × 10^−46^

^*^The nonfunctional alleles mean A allele of R90H or W258X.

^†^2 × 3 Fisher’s exact test.

**Table 2:** Genotype distributions of *URAT1* nonfunctional variants in 3,305 males and risk ratio for hyperuricemia.

		Hyperuricemia	Control*	*P* value†	RR (95% CI)	Reciprocal RR (95% CI)
R90H	G/G	806	2,477			
	G/A	0	22			
	A/A	0	0	4.3 × 10^−3^	—	—
W258X	G/G	804	2,347			
	G/A	2	150			
	A/A	0	2	3.3 × 10^−16^	0.041 (0.010–0.164)‡	24.5 (6.1–98.7)‡
Number of nonfunctional alleles(R90H or W258X)	0	804	2,325			
	1	2	172			
	2	0	2	6.7 × 10^−19^	0.036 (0.009–0.143)§	28.1 (7.0–112.8)§

3,305 males (806 hyperuricemia and 2,499 controls) are health examination participants of the J-MICC study.

Abbreviation: RR = risk ratio; CI = confidence interval.

^*^Control group is comprised of individuals with serum uric acid levels ≤ 7.0 mg/dl, no gout history and no treatments for gout/hyperuricemia.

^†^3 × 2 Fisher’s exact test.

^‡^Dominant model (G/G versus G/A or A/A).

^§^Dominant model (0 versus 1 or 2).

**Table 3:** Multiple regression analysis focused on the interaction between *URAT1* variants and sex.

	Partial regression coefficient	*P* value
*b*_*1*_	−2.21	8.2 × 10^−155^
*b*_*2*_	−1.72	<1.0 × 10^−323*^
*b*_*3*_	1.05	1.5 × 10^−12^

*y* = *b*_*0*_ + *b*_*1*_* *x**_*1*_ + *b*_*2*_* *x**_*2*_ + *b*_*3*_*x*_*1*_* *x**_*2*_, where *y* is SUA level, *x*_*1*_ is an ordinal variable representing the number of nonfunctional alleles of two *URAT1* variants (R90H and W258X), and *x*_*2*_ is a dummy variable representing the sex (male = 0 and female = 1). *x*_*1*_* *x**_*2*_ is an interaction term.

^*^P value was extremely low and the calculation was impossible by the software R.
