# Supplementary material for: The effects of URAT1/SLC22A12 nonfunctional variants,R90H and W258X, on serum uric acid levels and gout/hyperuricemia progression
Source: Sci Rep. 2016 Jan 29;6:20148. doi: 10.1038/srep20148 (PMC4731750; doi:10.1038/srep20148)
Supplement: Supplementary Information [file srep20148-s1.pdf]

SUPPLEMENTARY INFORMATION

The effects of *URAT1/SLC22A12* nonfunctional variants, R90H and W258X, on serum uric acid levels and gout/hyperuricemia progression

Masayuki Sakiyama, Hirotaka Matsuo*, Seiko Shimizu, Hiroshi Nakashima, Takahiro Nakamura, Akiyoshi Nakayama, Toshihide Higashino, Mariko Naito, Shino Suma, Asahi Hishida, Takahiro Satoh, Yutaka Sakurai, Tappei Takada, Kimiyoshi Ichida, Hiroshi Ooyama, Toru Shimizu & Nariyoshi Shinomiya

*Corresponding author. E-mail: hmatsuo@ndmc.ac.jp

Supplementary Table S1 | Genotype distributions of *URAT1* nonfunctional variants in 1,597 females

Supplementary Table S2 | Clinical characteristics of participants in the case-control study of gout

Supplementary Table S3 | Clinical characteristics of 4,902 participants evaluated for the effect of *URAT1* nonfunctional variants on serum uric acid

Supplementary Table S1 | Genotype distributions of *URAT1* nonfunctional variants in 1,597 females

		Hyperuricemia	Control[*]
R90H	G/G	24	1,568
	G/A	0	5
	A/A	0	0
W258X	G/G	24	1,512
	G/A	0	58
	A/A	0	3
Number of nonfunctional alleles[†]	0	24	1,507
(R90H or W258X)	1	0	63
	2	0	3

1,597 females (24 hyperuricemia and 1,573 controls) are health examination participants of the J-MICC study.

Abbreviations: SUA = serum uric acid.

[*]Control group is comprised of individuals with serum uric acid levels ≤ 7.0 mg/dl, no gout history and no treatments for gout/hyperuricemia.

[†]Nonfunctional alleles mean A allele of R90H or W258X.

Supplementary Table S2 | Clinical characteristics of participants in the case-control study of gout

	Gout	Control
Number	1,993	2,499
Age (year)	44.4 ± 11.8	52.8 ± 8.6
Body-mass index (kg/m^2)	25.1 ± 3.5	23.2 ± 2.7
Serum uric acid level (mg/dl)	8.5 ± 1.2	5.7 ± 0.9

Plus-minus values are means ± SD.

Supplementary Table S3 | Clinical characteristics of 4,902 participants evaluated for effects of *URAT1* nonfunctional variants on serum uric acid

	Males	Females
Number	3,305	1,597
Age (year)	52.8 ± 8.6	51.2 ± 8.6
Body-mass index (kg/m^2)	23.5 ± 2.9	22.0 ± 3.1
Serum uric acid level (mg/dl) *	6.1 ± 1.2	4.4 ± 1.0

4,902 individuals (3,305 males and 1,597 female) are health examination participants of the J-MICC study. Plus-minus values are means ± SD.

*Serum uric acid levels were analyzed in 4,753 individuals (3,158 males and 1,595 females), who received no medication for gout and/or hyperuricemia.
